# The Fibularis Quartus Muscle: A Cadaveric Case Report with Historical, Embryological, Molecular and Clinical Considerations

**DOI:** 10.15388/Amed.2024.31.2.12

**Published:** 2024-12-04

**Authors:** Dibakar Borthakur, Rajesh Kumar, G R Namaschivayam, Mohammed Ahmed Ansari, Seema Singh

**Affiliations:** 1 Anatomy, All India Institute of Medical Sciences, New Delhi, India; 2 Anatomy, All India Institute of Medical Sciences, Patna, India

**Keywords:** Fibularis quartus, Peroneus quartus, Peroneal compartment, Raktažodžiai: Fibularis quartus, Peroneus quartus

## Abstract

**Introduction:**

The fibularis quartus (FQ) or peroneus quartus (PQ) is a supernumerary muscle occasionally seen in the lateral compartment of the human leg. It is a weak evertor and has a role in pronation and lateral stabilization of the foot. FQ arises from the fibularis brevis (FB) in most instances and distally gets attached to the lateral aspect of the base of the fifth metatarsal or the cuboid or the calcaneus bone. The muscle has been implicated in a variety of clinical problems ranging from ankle pain to subluxation and tear of peroneal tendons. We report here a rare bitendinous variant of FQ which might provide new insight to clinical problems related to the presence of this muscle and its possible utility as an autograft in reconstruction.

**Methods:**

Institutional guidelines for use of human cadaver were followed. Routine dissection was performed on a 71-year-old male donated cadaver in both the lower limbs. Gross anatomical features were meticulously noted, photographed and measurements were recorded with digital Vernier callipers.

**Results:**

A bitendinous muscle was observed in the lateral compartment of the left leg and identified as FQ. The FQ was proximally attached to the FB and adjacent distal third of the fibula, which then formed a flat fusiform muscle belly and eventually terminated through a rounded tendon via its attachment to the peroneal trochlea of the calcaneus. The dimensions of the proximal tendon, muscle belly and the distal tendon were 4.2 cm x 0.7 cm, 5.6 cm x 1.9 cm and 2.6 cm x 0.3 cm, respectively.

**Conclusions:**

Clinicians should be aware about all possible variants of the supernumerary FQ muscle considering its role in several lateral ankle pathologies. FQ can prove as an excellent autograft for peroneal tendon tear and can be utilized for strengthening weakened peroneal tendons.

## Abstract (it)

**Įvadas:**

Keturgalvis raumuo (FQ arba PQ) – tai raumuo, kartais pasitaikantis žmogaus kojos šone. Jis yra silpnas raumuo ir atlieka pronacijos ir šoninės pėdos stabilizavimą. Dažniausiai FQ kyla iš *fibularis brevis* (FB) ir distaliai prisitvirtina prie penktojo metatarsalio pagrindo, kuboidinio arba kulnakaulio kaulo šoninės pusės. Raumuo yra susijęs su įvairiomis klinikinėmis problemomis, pradedant čiurnos skausmu ir baigiant peronealinių sausgyslių subliuksacija ir plyšimu. Čia pateikiame retą bitendininį FQ variantą, kuris gali suteikti naujų žinių apie klinikines problemas, susijusias su šio raumens buvimu, ir galimą jo kaip autotransplantato panaudojimą rekonstrukcijai.

**Metodai:**

Laikytasi institucijos rekomendacijų dėl žmogaus lavono naudojimo. Buvo atlikta įprastinė 71 metų amžiaus vyro dovanoto lavono abiejų apatinių galūnių skrodimo operacija. Bendrieji anatominiai požymiai kruopščiai užfiksuoti, nufotografuoti, o matavimai užrašyti skaitmeniniais *Vernier* stabilizatoriais.

**Rezultatai:**

Kairės kojos šone buvo pastebėtas bitendinis raumuo, kuris identifikuotas kaip FQ. FQ proksimaliai buvo prisitvirtinęs prie FB ir gretimo distalinio blauzdikaulio trečdalio, kuris vėliau suformavo plokščią susiliejantį raumens pilvą ir galiausiai baigėsi apvalia sausgysle per prisitvirtinimą prie kulnakaulio peronealinės sausgyslės. Proksimalinės sausgyslės, raumens pilvo ir distalinės sausgyslės matmenys buvo atitinkamai 4,2 cm×0,7 cm, 5,6 cm×1,9 cm ir 2,6 cm×0,3 cm.

**Išvados:**

Gydytojai turėtų žinoti apie visus galimus viršgumburinio FQ raumens variantus, atsižvelgiant į jo vaidmenį kelių šoninių kulkšnių patologijų atveju. FQ gali būti puikus autotransplantatas peronealinės sausgyslės plyšimo atveju ir tinkamas naudoti silpnoms peronealinėms sausgyslėms stiprinti.

## Introduction

The fibularis quartus (FQ) muscle also known as the peroneus quartus (PQ) muscle is a supernumerary muscle occasionally encountered in the lateral compartment of the human leg with a reported incidence of up to 22% [1,2]. Its exclusive occurrence in humans is presumably related to the adoption of bipedal gait [3]. When present, the muscle usually arises from the fibularis brevis (FB) and distally gets attached onto the base of the fifth metatarsal or the calcaneus bone [4–6]. The muscle is known by various names based on the origin-insertion pattern such as: peroneus accessorius, peroneus calcaneus externus, peroneocuboideus etc. [1,5]. It is believed to function as a weak evertor and probably has some role in lateral stabilization of the foot [7].

Embryology: The embryonic dorsal muscle mass formed from the myogenic precursors that migrate to limb buds during the 5th week of intrauterine life and contribute to the limb musculature. The limb muscles develop from the ventrally moving somites and innervated by ventral rami of the corresponding spinal nerves. Myogenic precursor cells start migrating to the developing lower limb bud at the 5th week of development and form two large condensations of muscle masses on the dorsal and the ventral aspects of the lower limb bud. The muscles of the lateral compartment of the leg arise from the dorsal muscle mass of the lower limb bud. The tendons of the limb develop from lateral plate mesoderm. Migration of muscle progenitors and delamination in the final environment takes place under the influence of several transcription factors. Important ones include Pax3, c-Met, Hgf, and Lbx1. C-Met expressing cells in the somitic dermomyotome delaminate and migrate in response to Hgf signalling which in turn is regulated by Pax3 [8]. It has been seen that limb muscles fail to develop in the Splotch mouse (a Pax3 mutant mouse), which reiterates the role of Pax3 in limb development. Lbx1 is another essential transcription factor absence of which results in inappropriate delamination and migration of limb muscle [9]. Full range of normal limb muscle development requires an appropriate number of myogenic cells which is under the control of a homeobox gene Meox2. Variant fibularis or peroneal muscles are believed to be due to deletion of Meox2 homeobox gene [9].

The FQ muscle has been implicated in several clinical problems in the ankle region. Mere presence of FQ is not worrisome, but occasionally the same can be the cause of lateral ankle pain, subluxation of the peroneal tendons, peroneal tendon tear, edema, etc. [1]. Clinical conditions secondary to the occurrence of the additional muscle can range from chronic pain in the ankle to tear and subluxation of the peroneal tendons [10,11]. Fibularis longus (FL) and FB tendons occupy the peroneal tunnel under the superior peroneal retinaculum [12]. Tendon of FQ in the narrow peroneal tunnel causes overcrowding, compromises the space and thus may cause clinical problems [13]. The FQ can be readily detected in magnetic resonance imaging (MRI) and ultrasonography. In ultrasonogram FQ is identified medially to the FB tendon with an intervening layer of loose areolar tissue [14,15]. Sometimes, missed FQ in imaging studies is detected intra-operatively during surgical exploration in the lateral compartment. A rare case of bitendinous FQ muscle originating from the FB and inserting on the peroneal trochlea of the calcaneus is presented here.

## Case Report

**Fig 1:**
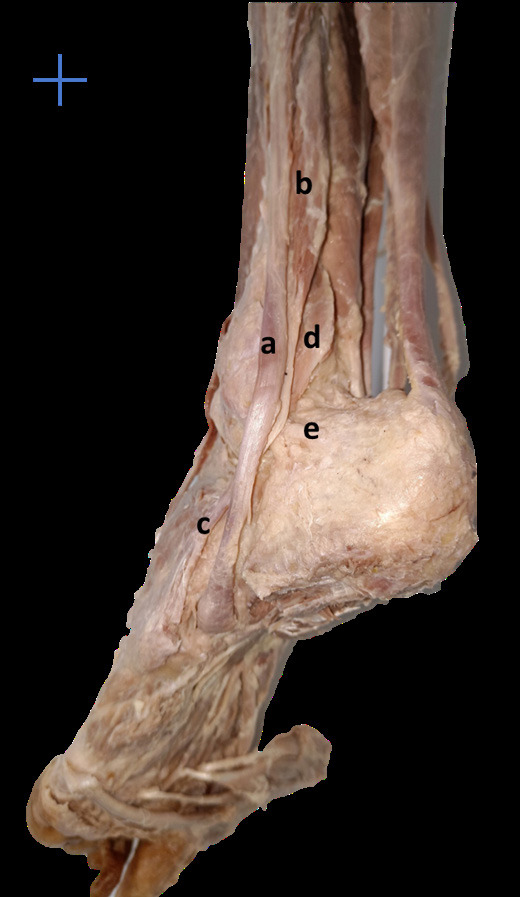
Muscles of lateral compartment of leg (a – fibularis longus, b – muscular part of fibularis brevis, c – tendinous part of fibularis brevis, d – muscular part of fibularis quartus, e – calcaneofibular ligament).

**Fig 2:**
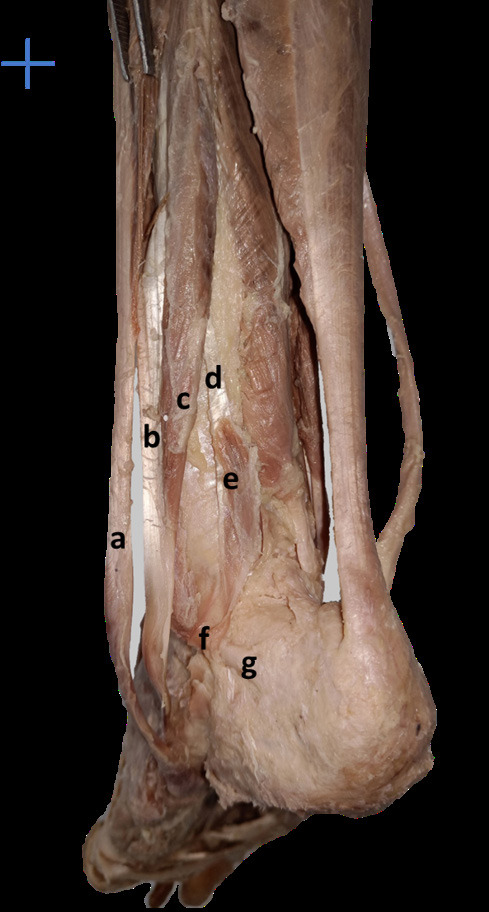
Tendon of fibularis quartus passing over calcaneofibular ligament (a – fibularis longus, b – tendinous part of fibularis brevis, c – muscular part of fibularis brevis, d – tendinous part of fibularis quartus, e – muscular part of fibularis quartus, f – fibularis quartus passing over calcaneofibular ligament, g – calcaneofibular ligament).

The FQ muscle was observed during routine dissection in the left leg of a 71-year-old male cadaver. In the lateral compartment of the left leg, a bitendinous muscle was observed partially under cover of the FL and FB muscles (Fig 1). The muscle originated from the FB and the adjacent middle third of the fibula as a flat tendon which then formed a muscle belly and eventually terminated as a rounded tendon which run superficial to the calcaneofibular ligament and got inserted on the posterior aspect of the peroneal trochlea on the lateral aspect of the calcaneus. Though the term origin and insertion is obsolete nowadays, the same has been retained in the article for the ease of description and understanding. The proximal and the distal tendon of the muscle were 4.2 cm x 0.7 cm and 2.6 cm x 0.3 cm, respectively. The flat fusiform muscle belly measured 5.6 cm x 1.9 cm in its widest dimension (Fig 2). The muscle belly also took attachment from the lower third of the fibula and the posterior intermuscular septum of the left leg. While traversing through the peroneal tunnel beneath the superior peroneal retinaculum, it was lodged in its own independent compartment with separate synovial sheath. The FL runs into the medial aspect of the sole under the cuboid tunnel, FB inserted on the base of the fifth metatarsal bone by multiple slips (Fig 2). The right leg had no accessory or supernumerary muscle.

## Discussion

Two supernumerary muscles of the lateral compartment of the leg have been commonly described in medical literature – the peroneus quartus or the fibularis quartus and the peroneus digiti quinti. Otto and colleagues first described FQ in 1816 [16]. Wood in 1868 stated that FQ replaced the FB, and had the opinion that FQ may insert onto the cuboid and thereby can be a variant of peroneus tertius muscle [17]. Gruber in 1884 systematically studied the muscle in cadavers and documented 13% prevalence of the FQ at that time. In 1974, White and his colleagues opined that FQ originates from the lower third of fibula and inserts on the calcaneus [18]. The reported prevalence of FQ varies from 6.6% to 22% in cadaveric studies and up to 17% in imaging studies [11,19]. No definite race or gender predilection has been linked. However, Cheung et al. in 1997 opined that FQ occurrence was more common in male. A recent study documented almost equal prevalence of FQ in both genders (19). Right sided FQ has been observed more frequently in literature. Occasionally bilateral occurrence of the FQ has also been reported [19]. Otto in 1816 stated that FQ originated from the lower third of the fibula and runs distally to the lateral aspect of the calcaneus [18]. Biligili et al. reported the proximal attachment of the muscle to be mostly from the FB muscle [11]. Furthermore, they also observed that the FQ occurrence was more common in instances where the FB muscle itself was less well developed [11,20]. This fact indicates that the FQ can be a fragmented muscle of the FB which got separated during evolution. In the present case of the bitendinous FQ on the left leg, proximally it was attached to the FB and the adjacent fibula through its flat tendon and distally the muscle anchored onto the peroneal trochlea of the calcaneus bone through a rounded tendon. The trochlear, retrotrochlear and several other tendinous attachment patterns of the FQ have been described in erstwhile literature [11], but a bitendinous FQ has not been described yet. The muscle belly of the FQ was separated all throughout the lateral compartment from the muscle bellies of the FB and FL. The present case had FQ originating from FB, adjacent fibula and the posterior intermuscular septum similar to the description by Athavale et al. and Inchai C et al. [16] and from the muscle resembles closely with the type VI variety according to the classification system proposed by Hur M S et al. [21]. The rate of prevalence of FQ in cadaveric and imaging studies and highlights of some important clinical cases is presented in Table 1.

**Table 1:** Prevalence of FQ in some recent cadaveric and imaging studies and highlights of some important clinical cases on FQ.

Authors	Sample size	Population and type of study	Notable findings	Other remarks
Cossogue et al., 2023 [22]	3	American, Clinical case	FQ tendon was successfully used for the repair of FB tendon tear in three patients.	2 females, 1 male, all right sided.
Kobayashi et al., 2023 [24]	1	Japanese, Clinical case	FQ presenting as severe leg pain in a 21 year old male.	
Inchai et al., 2021 [16]	60	Thai, Cadaveric study	6.67% prevalence of FQ.	All the FQ were noted unilaterally on the left side, gender unspecified.
Lui and Li, 2020 [25]	1	Chinese, Clinical case	Describe a symptomatic FQ and detailed the procedure of endoscopic resection.	Left sided, gender unspecified.
Sperle et al., 2019 [26]	1	American, Clinical case	FQ presenting as chronic left ankle pain following trauma.	FQ undetected in routine imaging studies, but detected intra-operatively
Dangintawat et al., 2018 [27]	109	Thai, Cadaveric study	12% (7% male and 5% female) prevalence of FQ.	FQ noted bilaterally in two cases (1 male, 1 female). Retrotrochlear eminence was the most common insertion site.
Habashy et al., 2017 [1]	1	American, Clinical case	FQ presented as right ankle pain in a 16-year old boy which was associated with subluxation of FQ tendons.	Clinical symptoms subsided after resection of FQ
M S Somesh at al., 2016 [28]	47	Indian, Cadaveric study	4.26 % (2 out of 47) unilateral prevalence.	FQ was observed in two cases on the left side.
Grace, Ramaya and TSR, 2016 [29]	64	Indian, Cadaveric study	1.56 % unilateral prevalence of FQ.	
Opdam K T et al., 2016 [30]	1	Dutch, Clinical case	Unexplained clicking and locking phenomenon of the right ankle in a 64 year old female due to FQ treated by resection.	
Yammine, 2015 [31]	3928	Meta-analysis	Overall FQ incidence of 10.2% and higher prevalence was observed in Indians.	
Bilgili, Kaynak, Botanlioglu et al., 2013 [6]	115	Turkish, Cadaveric study	5.2% prevalence of FQ and FQ absence was found associated with less developed FB	There was no correlation between presence of FQ and height of retrotrochlear eminence.
Clarkson et al, 2013 [32]	277	American, Cadaveric study	20.9% prevalence of PQ.	Described two subtypes of FQ and coined those peroneocuboideus and peroneocalcaneocuboideus
Athavale et al., 2012 [33]	92	Indian, Cadaveric study	21% unilateral prevalence of FQ.	FQ was present in 9 right and 11 left sided legs.
Tubbs et al., 2008 [17]	1	American, Clinical case	Described a unique muscle and termed it Peroneotalocalcaneus and postulated that this could be possible variation of FQ muscle.	
Zammit and Singh, 2003 [2]	102	British, Cadaveric + Radiologic	6.6% prevalence of FQ.	Sidedness and gender unspecified.
Oznur, Arik and Alanay, 2002 [34]	1	Turkish, Clinical case	FQ was identified as a rare cause of chronic ankle pain	
Chepuri et al, 2001 [14]	7	American, Clinical case series	The FQ muscle appeared hypoechoic, while the tendon of the FQ appeared hyperechoic and fibrillar.	

FQ congests the lateral compartment usually occupied by FL and FB tendons [10] and thus produces several lateral ankle pathologies and pathologies of the lateral compartment of the leg such as peroneal tendon split, tendinous calcification and painful hypertrophy of the retrotrochlear eminence, etc. [19]. Occasionally the tendon of FQ may weaken and undergo longitudinal tear thereby producing symptoms [20]. Zammit and Singh, 2003, while studying the MRI images of the 80 patients with lateral ankle problems could identify the FQ muscle in 6.6% cases. They also found FQ to be associated with tear and dislocations of the other peroneal tendons [19]. Early in 1974, White et al. attributed chronic ankle pain due to FQ muscle and postulated that the FQ muscle may mechanically impinge on the peroneal retinaculum thereby causing ischaemia, inflammation and pain. Sobel et al. in their study demonstrated two-fold increases in the risk of FB attrition when a concomitant FQ was present. Wenning et al. noted that FQ is associated with chronic exertional compartment syndrome. Although FQ related clinical problems has been reported both in young as well as elderly age group, no definite association with age has been observed yet. More importantly, the FQ has the potential to be harvested as an autograft for use in reconstructive surgeries for fixing other peroneal tendons. In a recently published case series in three patients, torn FB tendons were successfully repaired with FQ autograft. None of those three patients reported any adverse events after the removal of the FQ such as gait alterations or decrease in ankle strength [22]. FQ has also been used for reconstruction of the peroneal retinaculum [23]. In the present case, we could not find any evidence of the clinical problem due to the FQ while the person was alive. Probably, the muscle was quiescent and not congesting the peroneal compartment in the deceased.

Symptomatic FQ can be readily detected with MRI and ultrasonography. The appearance of the FQ in ultrasound images varies from case to case. Typical sonographic appearance of the FQ has been described as a discrete structure located posteromedial or medial to the FB and FL muscles in the lateral compartment and of the same echotexture with that of FB or FL muscle but hypoechoic relative to adjacent loose soft tissue. The tendon part of the FQ appears hyperechoic [14]. FQ as a cause of chronic lateral ankle pathology is less commonly suspected. However, it is recommended that if chronic lateral ankle pain is unresponsive to conventional standard treatment, the presence of FQ muscle should be considered in the differentials and thoroughly investigated preferably with imaging studies. Symptomatic FQ is best diagnosed with MRI and excision is the treatment of choice.

## Conclusions

Fibularis quartus muscle might remain quiescent for the lifetime, but occasionally it can be the hidden cause for chronic lateral ankle pain, lateral ankle instability, swelling, peroneal tendon tear, etc. FQ can be mistaken for peroneal tendon tear. When such chronic conditions are not attributable to any local aetiology even after thorough investigations, the possibility of the presence of FQ should be considered in the differential diagnosis.

## Limitations of the study

The present report is based on the observation of a rare variant muscle unilaterally, i.e. FQ in one cadaveric case. The bitendinous variety of FQ is rare and seems to represent a novel variant to be introduced to clinicians. But the findings cannot be generalized and may not apply to other individuals. The clinical relevance and utility of the FQ that has been discussed in the article is entirely based on existing literature, and not based on the observations made on an individual where FQ was present, which is another shortcoming of the report.

## Abbreviations used

PQ –peroneus quartus

FQ –fibularis quartus

FB –fibularis brevis

FL –fibularis longus

MRI –magnetic resonance imaging

Pax3 –paired-box transcription factor

c-Met –mesenchymal-epithelial transition factor, a proto-oncogene,

Hgf –hepatocyte growth factor

Lbx1 –homolog of the Drosophila lady bird late gene

Meox2 –mesenchyme homeobox 2, a homeobox gene; also called Mox2
